# Simulations on the dual effects of flavonoids as suppressors of Aβ42 fibrillogenesis and destabilizers of mature fibrils

**DOI:** 10.1038/s41598-020-72734-9

**Published:** 2020-10-06

**Authors:** Sahar Andarzi Gargari, Abolfazl Barzegar

**Affiliations:** 1 Research Institute of Bioscience and Biotechnology, University of Tabriz, Tabriz, Iran; 2 Department of Medical Biotechnology, Faculty of Advanced Medical Sciences, Tabriz University of Medical Sciences, Tabriz, Iran

**Keywords:** Computational biology and bioinformatics, Computational models, Computational neuroscience, Protein structure predictions, Computational chemistry, Proteins, Small molecules

## Abstract

Structural studies of the aggregation inhibition of the amyloid-β peptide (Aβ) by different natural compounds are of the utmost importance due to their great potential as neuroprotective and therapeutic agents for Alzheimer’s disease. We provided the simulation of molecular dynamics for two different states of Aβ42, including “*monomeric* aggregation-prone state (APS)” and “U-shaped pentamers of amyloidogenic protofilament intermediates” in the absence and presence of polyphenolic flavonoids (Flvs, myricetin and morin) in order to verify the possible mechanism of Flvs fibrillogenesis suppression. Data showed that Flvs directly bind into Aβ42 species in both states of “monomeric APS β-sheets” and “pentameric amyloidogenic intermediates”. Binding of Flvs with amyloidogenic protofilament intermediates caused the attenuation of some inter-chains H-bonds, salt bridges, van der Waals and interpeptide interaction energies without interfering with their secondary β-sheets. Therefore, Flvs redirect *oligomeric* amyloidogenic intermediates into unstructured aggregates by significant disruption of the "steric zipper" motif of fibrils—pairs of self-complementary β-sheets—without changing the amount of β-sheets. It is while Flvs completely destruct the disadvantageous secondary β-sheets of *monomeric* APS conformers by converting them into coil/helix structures. It means that Flvs suppress the fibrillogenesis process of the monomeric APS structures by converting their β-sheets into proper soluble coil/helices structures. The different actions of Flvs in contact with two different states of Aβ conformers are related to high interaction tendency of Flvs with additional H-bonds for monomeric APS β-sheet, rather than oligomeric protofilaments. Linear interaction energy (LIE) analysis confirmed the strong binding of monomeric Aβ-Flvs with more negative ∆G_binding,_ rather than oligomeric Aβ-Flvs system. Therefore, atomic scale computational evaluation of Flvs actions demonstrated different *dual* functions of Flvs, concluded from the application of two different monomeric and pentameric Aβ42 systems. The distinct *dual* functions of Flvs are proposed as suppressing the aggregation by converting β-sheets of monomeric APS to proper soluble structures and disrupting the "steric zipper" fibril motifs of *oligomeric* intermediate by converting on-pathway into off-pathway. Taken together, our data propose that Flvs exert dual and more effective functions against monomeric APS (fibrillogenesis suppression) and remodel the Aβ aggregation pathway (fibril destabilization).

## Introduction

**Figure 1:**
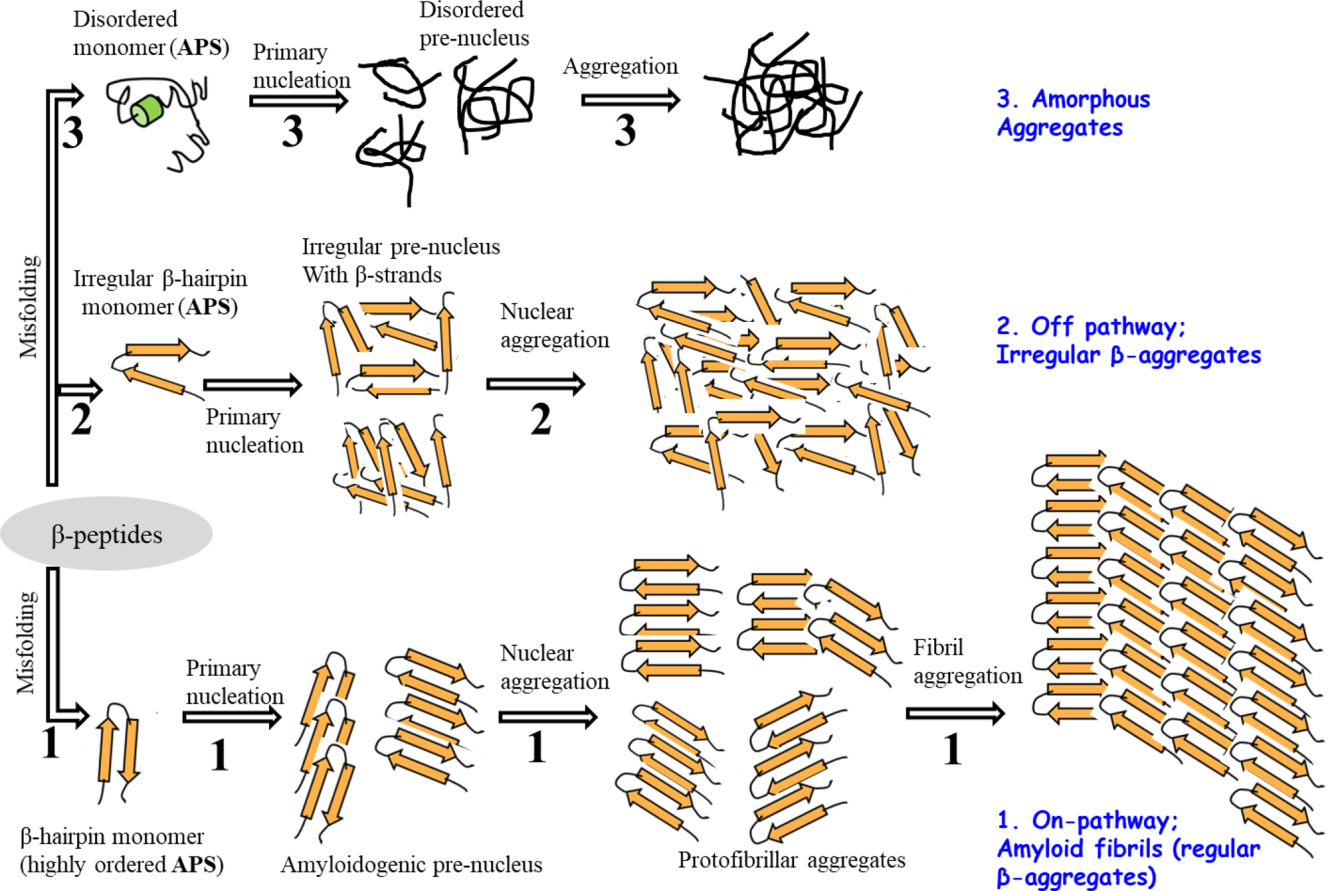
Schematic illustration of protein aggregation through misfolding with different pathways. Native β-peptides misfold and undergo conformational changes in aggregation-prone state (APS) by forming highly ordered β-hairpin monomers, irregular β-hairpin monomers and disordered monomers. Nucleation of different APS conformers results in pre-nucleus oligomers that end in different aggregates, as on-pathway fibrils (Path **1**), off pathway aggregates (Path **2**) and amorphous aggregates (Path **3**).

Alzheimer’s disease (AD) is the most common progressive memory loss and the leading cause of dementia in elderly people, which currently affects more than 12 million people worldwide^1^. One of the major pathological hallmarks of AD is the aggregation and accumulation of amyloid beta (Aβ) peptides in neural tissues of the brain^2^. Generation of Aβ peptides involves sequential cleavage of β and γ secretases from amyloid precursor protein (APP)^3^. γ-secretases produce two most common forms of peptides, namely Aβ40 and Aβ42, with 40 and 42 amino acid residues, respectively. Aβ42 is the most abundant form in the disease state and the more readily formed aggregate in solution, that is associated with increased protofibrils neurotoxicity^4^. Therefore, AD is associated with the production of Aβ peptide, followed by Aβ aggregation in the brain. Self-assembling forms of Aβ peptides lead to the formation of βsheet-rich oligomers and larger plaques^5^. Consequently, the aggregation of Aβ peptides finally ends in the formation of insoluble protofibrils/fibrils as components of amyloid plaques. The possible mechanism for misfolding and oligomerization of Aβ peptides into different types of aggregates is illustrated in Fig. 1, which includes amyloid fibrils, irregular β-aggregates and amorphous unstructured aggregates. These phenomena most likely occur through three different pathways, comprised of specific on and off-pathways and a non-specific amorphous path^6^. In the on-pathway chemical process, oligomers end in fibril components of amyloid plaques through infinite polymerization degree of highly ordered intermediates state, derived from monomeric aggregation-prone state (APS) β-sheet structures. In the model showed in Fig. 1, the misfolded regular U-shaped β-strand of APS monomers form oligomers of pentameric protofilaments at critical concentration that ultimately result in mature amyloid toxic fibrils via on-pathway mechanism through the primary nucleation process. On the other hand, the off-pathway is another specific chemical process that converts irregular APS β-hairpin monomers to oligomeric aggregate through a finite degree of polymerization, which do not terminate to toxic fibrils. Another possible non-specific pathway is related to misfolding and oligomerization of disordered coil monomers into tangled amorphous and unstructured aggregates. Since both the off-pathway chemical process and non-specific amorphous mechanism do not end in fibrils, the constructed aggregates are initially non-toxic for cells^6^. Therefore, diverting on-pathway Aβ aggregation into off-pathway irregular β-aggregates and/or amorphous agglomerates is accompanied by suppression of the Aβ fibrils formation. This is one of the main challenges and beneficial strategies to apply for neuroprotective and therapeutic agent in Alzheimer’s disease. In this regard, fibrillogenesis of Aβ peptides has been studied by various experimental and theoretical methods using plaque-binding antibodies^7^, by designing Aβ peptide-binding proteins^8^, and through applying non-peptidal small molecules^9^ and natural compounds with anti-amyloidogenic properties^10,11^. Natural compounds, such as flavonoids and polyphenols found within fruits and vegetables have been shown to reduce the risk of AD and to have protective effects in inhibiting Aβ aggregation and destabilization of fibrils^12^. In vitro findings demonstrate a physical interaction between flavonoids and Aβ peptides that culminate in reducing Aβ fibrils’ levels. Polyphenols have tendency to interact with β-sheet structures of Aβ peptides that inhibit Aβ aggregation and destabilize the formed fibrils^11,13^. Therefore, the anti-aggregation properties of polyphenols are mediated by direct interaction with amyloid peptide, and especially by interference with β-sheet structures^14^. Although fibrillogenesis of the Aβ24 has been extensively studied, the accurate mechanism remains unclear regarding the on-pathway prevention of Aβ aggregation and/or redirection into off-pathway. Computational methods are useful to readily investigate the oligomerization mechanism of misfolded Aβ peptides and to develop any effective additive that inhibits the corresponding pathways. Thus, in this research, we performed computational MD simulations of Aβ42 in both monomeric APS and pentameric protofilament intermediate states (pre-nucleus aggregates), following their possible physical and structural changes in binding with two known flavonoids (Flvs) of myricetin and morin. Our findings revealed dual different functional behavior of Flvs, in case they are exposed to be in contact with APS monomers or pre-nucleus aggregates. Flvs are more effective against APS of Aβ peptides with inhibiting the primary nucleation process by recovering their coil conformation. For amyloidogenic pre-nucleus aggregates of pentamers, Flvs redirect the on-pathway Aβ fibrils into off-pathway aggregates.

## Materials and methods

### Simulation setup

Since there are no high-resolution data on the structure of the physiologically relevant or "most toxic" oligomeric aggregates of Aβ42 so far, the human U-shaped pentameric protofilament structure (PDB code 2BEG) was taken as a model system to follow the on-pathway fibrillogenesis^15^. In this structure, the core region of β-sheet structures, including 17–42 residues of each Aβ peptide, are highly ordered in pentameric state as a popular model of intermediate conformers of fibril system in regard to on-pathway process^16^. The detailed structural features of the selected U-shaped pentamer as an intermediate of fibril-like protofilament model with cross β structures are displayed in Supplementary Fig. S1. By focusing on specific and distinctly different effects of Flvs on APS monomers vs. pentameric protofibrils, we have extracted monomeric pdb structure with the same core region of β-sheet structures including 17–42 residues (monomeric β-hairpin) for additional studies. Therefore, the two applied monomeric and pentameric types of Aβ structures are directly related to “primary APS” and “amyloidogenic pre-nucleus aggregates via on-pathway mechanism”, respectively.

The initial structures of polyphenolic myricetin and morin flavonoids (Flvs) were generated and geometries were optimized using the semi-empirical AM1 followed by ab initio DFT method with Becke's three-parameter hybrid function^17–19^ (Fig. S2). Before performing molecular dynamic simulation (MD), the force field parameters for Flvs of myricetin and morin were achieved by PRODRG 2 server for using in MD simulations^20^ (https://davapc1.bioch.dundee.ac.uk/cgi-bin/prodrg). Moreover, the N-terminal ends of each peptide were previously acetylated in order to give uncharged N-termini. To study the Flvs dual possible functional effects, different simulation systems were prepared, including monomeric β-hairpin and U-shaped pentameric protofilament of Aβ42 in free state and in complex with myricetin and morin Flvs. We applied 10:1 mol ratio of Flvs/Aβ42 for complexes of both monomeric β-hairpin and pentameric protofilament systems. This mole ratio was applied in MD simulation studies for using natural and small excipient compounds against Aβ42 aggregation prohibition^16,21–24^. Flv molecules were randomly embedded inside the simulation box, separately dispersed in the solvent with at least 4 Å distance from each other and from the nearest atoms of Aβ peptides. The distances of 4 Å was applied to ensure that the initial steric clashes between Flv-Flv and/or Flv-Aβ peptide atoms won’t affect the MD simulation. This distance is beyond the interatomic physical bond lengths (mainly hydrogen bonds, < 3–3.5 Å) between the donor and acceptor atoms^17^. Therefore, the distance of 4 Å is enough to avoid any physical interaction between Flv-Flv and/or Flv-Aβ peptides before MD simulation at initial stage. All simulation parameters were generated by GROMACS 5.0.4 program package, applying the GROMOS96 53A6 force field^25^. The systems were separately solvated with an SPC water model that extends up to 10 Å from any edge of the cubic box up to the solute atoms. The NaCl concentration in all simulation systems was 100 mM, achieved by adding appropriate number of Na^+^ and Cl^−^ ions. In all cases, short-range non-bonded interactions were truncated at 1.2 nm applying long-range dispersion correction to the energy and pressure terms, in order to investigate truncation of the van der Waals interactions. The Particle Mesh Ewald (PME) method was utilized for the calculations of long-range electrostatic interactions. The LINCS algorithm^26^ was used for all bond constraints, allowing an integration time step of 2 fs. Periodic boundary conditions were applied in all directions. Temperature of the systems was preserved at 310 K by using Berendsen weak coupling method, while pressure was maintained at 1 bar by utilizing Parrinello-Rahman barostat in constant pressure ensemble. All systems were energy-minimized using the steepest descent method. The minimized systems were equilibrated under NVT (constant volume) and NPT (constant pressure) ensemble conditions, respectively, for 200 ps time scale. Visual Molecular Dynamic (VMD) software version 1.9^27^ was used to display the structural changes during the simulation runs.

### LIE setup for Aβ42-Flv binding free energy

Due to the great potential of neuroprotective and therapeutic agents for direct binding to Aβ42 species in amyloid mature fibrils, we have applied linear interaction energy approximation (LIE) method for estimating the Aβ peptides-Flv binding free energy (∆G). LIE is based on the direct pepetide-Flv interaction using the initial and final states of the binding in MD simulation process, which corresponds to the free and bound state of the ligand. This method statistically estimates the ∆G value by the average values of the electrostatic and van der Waals interactions between each residue of peptide and ligand Flv molecules^28^.

$$ \Delta {\text{G}} = \mathop \sum \limits_{i} w_{i}^{vdw} E_{i}^{vdw} + \mathop \sum \limits_{i} w_{i}^{ele} E_{i}^{ele} + c $$

where the interaction energies of the electrostatic and van der Waals terms are defined as $$E_{i}^{ele}$$ and $$E_{i}^{vdw}$$ between the ligand and the i-th residue of a target peptide, respectively, and c is a constant. The terms of $$w_{i}^{vdw} $$ and $$w_{i}^{ele}$$ are parameters that can be determined by partial least squares analysis. Simulations of free ligands in water are necessary for calculating protein/ligand binding affinities using LIE approach. Therefore, the additional MD simulations were performed for Flvs in water by similar procedure as described in the previous section.

### Umbrella sampling setup

The umbrella sampling simulation is a very useful approach to extract the potential of mean force (PMF) and to achieve the ΔG for the binding condition, especially for the protein–protein or peptide-peptide interactions (PPI)^29,30^. For this aim, the previously simulated structures were used as starting configurations for pulling additional simulations. Aβ protofibril structures were placed in a rectangular box with an adequate size to provide the space for pulling the chain A out of the protofibril structure along the *z*-axis. As before, systems were separately solvated with SPC water and neutralized by adding appropriate number of NaCl counterions. Equilibration was carried out for 200 ps under a constant pressure (NPT) ensemble using the same steps as described above. Following equilibration, chain A was pulled apart from the protofibril along the vector defined by its centers of mass (COMs) at a rate of 0.0015 nm/ps for 4000 ps, resulting in a further 4.0 nm separation. Chain B was restrained as a stable reference for the pulling simulations. The output from pulling calculation was then used for preparing umbrella sampling windows. Thirty-five windows, with a separation of 0.1–0.2 nm between the COM of chain A and B, were extracted on the dissociation pathway. For further equilibration, each window was equilibrated for 100 ps and then 5 ns was performed for umbrella sampling simulations. Results obtained from the US simulations were analyzed by the weighted histogram analysis method (WHAM)^29^ in order to extract the potential mean force (PMF).

### gRINN setup for residue interaction analyses

In order to characterize the residue-residue interaction energy data from each protofibril in MD simulation trajectories, a post-simulation analysis was performed by gRINN (get Residue Interaction eNergies and Networks) method^31^. gRINN is known as straightforward and stand-alone software for analyzing the pairwise amino acid interaction energy and supports the output simulation files generated by NAMD/GROMACS software. This software requires the files describing the protein structure (PDB/TPR), topology (TOP) and trajectory (TRR/XTC) as input files. Solvent and non-protein molecules must be removed from all input files prior to utilization. Generated data from 2500 MD simulation trajectories were used to extract the important residues interaction by energy-based analysis^30^.

## Results and discussion

### Flvs-Aβ42 binding in monomeric and protofilament state

#### H-bond formation possibility between Flvs-Aβ in monomeric and oligomeric forms

**Figure 2:**
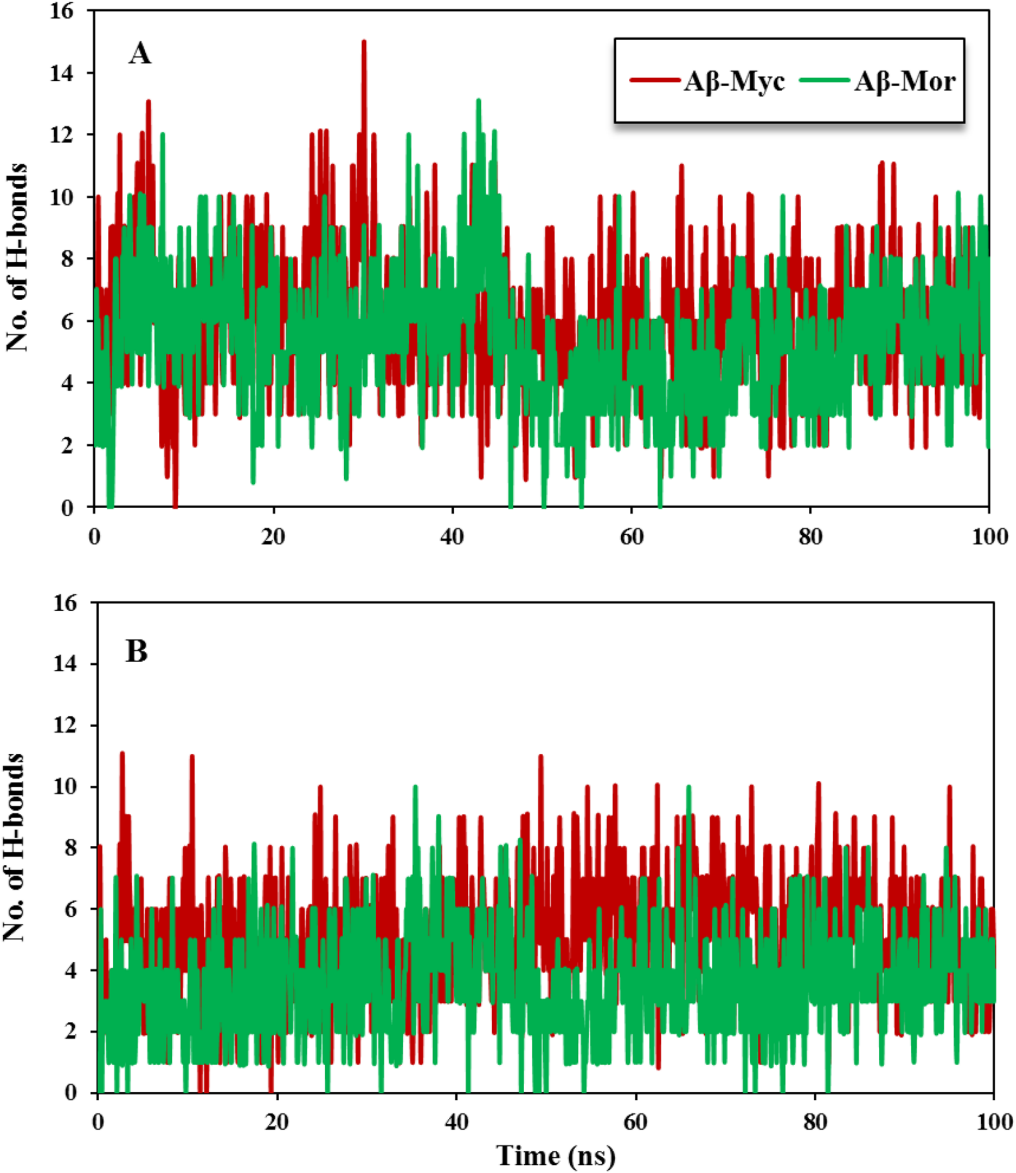
A number of H-bonds between Flvs with monomeric and oligomeric pentamer form of Aβ during the simulation time. (**A**) Flvs-monomeric Aβ system. (**B**) Flvs-pentameric Aβ system.

Since the anti-aggregation properties of the drug candidates and natural small compounds are mediated via direct binding with β-sheets structures^9–11,14^, analysis of the hydrogen bond (H-bond) pairings between Flvs and Aβ42 would be more efficient in determining Flvs-Aβ42 interactions. Flvs have several numbers of hydrogen bond acceptor and hydrogen bond donor groups, as shown in Supplementary Fig. S2. According to the experimental and theoretical studies, hydrogen-bonding interactions are necessary for protein/polyphenols strong association^32^. Therefore, the H-bond formation possibility analysis between Flvs and β-sheet structures of monomeric and oligomeric Aβ forms was performed during the simulation time in Fig. 2. This analysis clearly revealed that Flvs construct stable H-bonds with both monomeric and oligomeric Aβ forms. Myricetin and morin Flvs comprise 6 and 5 average H-bonds with monomeric Aβ forms, respectively. In addition, both myricetin and morin Flvs form 5 and 4 average H-bonds with oligomeric Aβ species, too. As a whole, Flvs have a high tendency to bind to both monomeric and oligomeric forms of Aβ. However, Flvs have high interaction tendency for additional H-bonding with monomeric APS β-sheet structure, compared to oligomeric U-shaped protofilaments. Therefore, it is expected that Flvs should be more effective against monomeric APS Aβ via powerful and direct binding interaction, relative to pre-nucleus aggregates of pentameric protofilament intermediates.

#### Linear interaction energy (LIE) analysis for Flvs-Aβ complexes

Computational prediction of protein–ligand binding affinity is utmost important in computer-aided drug designing, but performing powerful and accurate ∆G_binding_ computations is still challenging. Thus, estimation of binding affinities for protein–ligand complexes remains as the most important challenge in drug research^28^. In LIE, ∆G_binding_ is directly computed from differences in binding energies of ligand–surrounding between the free and bound states. It is believed that the predictive accuracy could be readily obtained for a small subset of compounds^33^. Since Flvs, as small subset compounds, bind to Aβ42 both in monomeric and oligomeric species, we have applied LIE method for estimating ∆G_binding_ in Aβ peptides-Flv complexes. This method statistically estimates the ∆G value by the average values of the electrostatic and van der Waals interactions between each residue of the peptide and the ligand Flv molecules^33,34^. The binding free energies of Flvs in different systems have been calculated and summarized in Table 1. The results indicate that both of myricetin and morin Flvs have the effective binding energy values for a given monomeric and oligomeric Aβ system. The ∆G_binding_ values of binding free energies are more negative for monomeric APS states of Aβ, compared to pre-nucleus oligomeric system (see ∆G*_binding_ in Table 1). The results are in good agreement with in vitro experimental total binding energy (∆Gexp) based on half maximal inhibitory concentration (IC_50_) of myricetin and morin with 15.1 μM and 30.3 μM, respectively^35^.

$$ \Delta \text{Gexp} = RT\,{\text{Ln}}\,{\text{IC}}_{50} $$

**Table 1:** Binding free energy averages of Flvs with monomeric and oligomeric pentamer form of Aβ peptide using semi-empirical LIE method, which is based on the average van der Waals (V^vdw^, nonpolar interactions modeled by Lennard–Jones potential) and the average electrostatic interactions energy (V^el^).

Peptides-Flvs	Ligand surrounding interactions						
	〈V^vdw^〉_bound_	〈V^vdw^〉_free_	〈V^el^〉_bound_	〈V^el^〉_free_	∆*G*_binding_	∆G*_binding_	∆*G*_*exp*_
Aβ_monomer_-Myc	− 220.7	− 139.3	− 144.8	− 143.6	− 15.95	− 15.95	–
Aβ_monomer_-Mor	− 208.3	− 131.5	− 140.7	− 616	− 11.35	− 11.35	–
Aβ_pentamer_-Myc	− 362.7	− 139.3	− 152.2	− 143.6	− 45.68	− 9.13	− 7.09
Aβ_pentamer_-Mor	− 342.9	− 131.5	− 618.1	− 616	− 39.63	− 7.92	− 6.65

The terms *bound* and *free* represent simulations of peptid-Flv-water complexes and free Flvs in water, respectively. ∆G*_binding_ as a corrected ∆*G*_binding_, corresponds to the average binding free energy of Flv per chain of pentameric Aβ peptide. ∆Gexp is based on in vitro half maximal inhibitory concentration of myricetin and morin Flvs. All energies are presented in kcal/mol.

Also, the results of ∆G_binding_ analyses support the previously discussed H-bond interactions with more potent monomeric Aβ-Flvs binding, relative to oligomeric Aβ-Flvs interactions.

### Secondary and 3D structural changes of Flvs-Aβ peptide complexes

**Figure 3:**
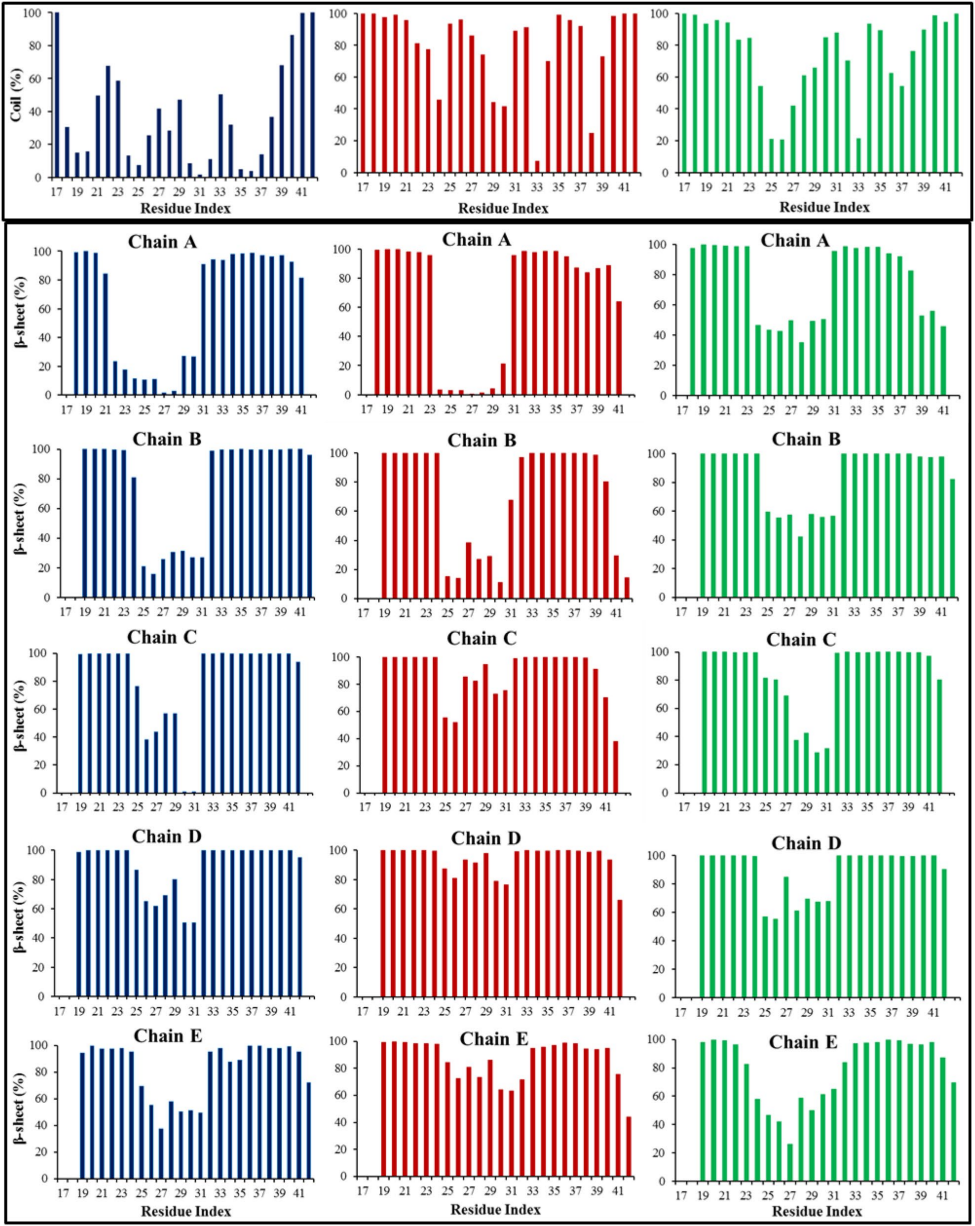
The secondary structure content of monomeric and oligomeric Aβ systems along the peptide sequence in Flv-free and Flvs-treated systems. Coil and β-sheet contents were evaluated for Aβ systems of monomeric (upper panel) and oligomeric (lower panel), respectively. Flv-free Aβ (blue;  ), Aβ in complexes with myricetin (red;  ) and morin (green;  ) Flvs.

Transition from secondary structure content into formation of β-sheet features is a crucial early step in neurotoxicity and Aβ amyloidogenesis. Aβ monomers with initial β-sheet structures self-assemble into oligomers and aggregate into toxic fibril forms via primary nucleation mechanism^4,5^. Therefore, evaluating the influence of Flvs on the β-sheet structure content of Aβ peptide in the given monomeric APS and pre-nucleus pentameric protofibril states would be very useful to understand the functional anti-aggregation and fibrillation suppressing effects of Flvs^11^. According to the secondary structure analysis in Fig. 3 and Fig. S3 by DSSP method^29^, β-sheets were completely disappeared by changing to coil structure in Flvs-treated monomeric Aβ systems (see also Table 2). It should be mentioned that transformation of the harmful secondary structure content into coil or α-helical features is an essential early step in preventing the amyloid fibrillation process through inhibiting primary nucleation. However, the structural changes in oligomeric Flvs-Aβ systems are negligible (Fig. 3 and Table 2). The secondary structure compositions of pentameric complex systems with Flvs are similar to Flv-free systems and maintain the β-strand conformation during the simulation time.

**Table 2:** The percentage values of different secondary structure contents of monomeric and oligomeric Aβ systems in complex with Flvs during 100 ns simulations.

Systems	% β-sheet	% Coil	% Bend/turn/helix
Aβ_monomer_	24	39	36
Aβ_monomer_-Myc	0	80	20
Aβ_monomer_-Mor	0	75	24
Aβ_pentamer_	72	19	5
Aβ_pentamer_-Myc	74	19	4
Aβ_pentamer_-Mor	75	18	4

All systems, including Flvs-free Aβ, Aβ in complex with myricetin (Aβ-Myc) and morin (Aβ-Mor) Flvs were analyzed and represented.

**Figure 4:**
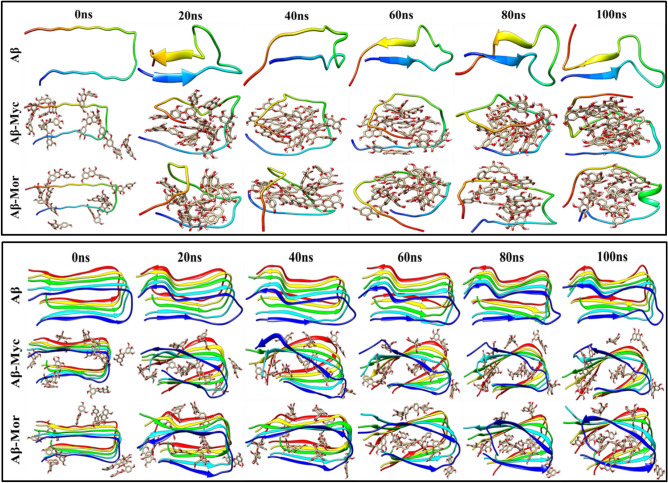
Different graphical 3D conformational snapshots of monomeric (upper panel) and pentameric protofibril (lower panel) forms of Aβ during 100 ns of MD simulations. Complex systems were initially prepared by placing Flvs on the surface of Aβ peptides with surrounding approximate distances of 4 Å; Flvs were dislocated inside the U-shaped peptide conformation during simulations. All systems, including Flv-free Aβ, Aβ in complexes with myricetin (Aβ-Myc) and morin (Aβ-Mor) Flvs are shown.

**Figure 5:**
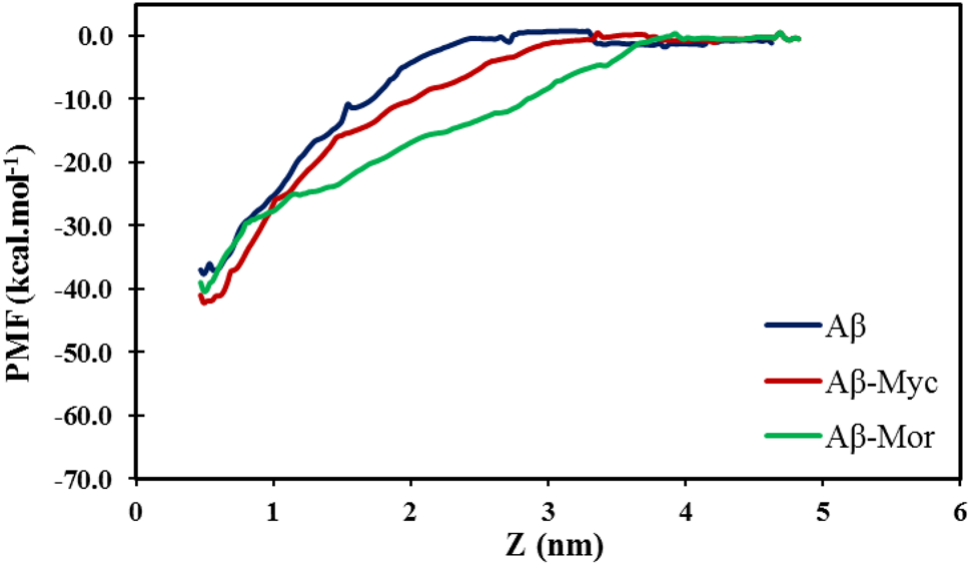
PMF profiles of chain A in the pentameric Aβ structure for Flv-free, as well as complex states with different Flvs based on umbrella sampling simulations.

Different structure snapshots in the MD simulations were prepared at every 20 ns, which clearly demonstrate the graphical representation of 3D conformational change possibility of Aβ peptides at Fig. 4. During simulation time, Flvs translocate inside the peptide conformation both in monomeric and oligomeric systems via direct binding with peptide backbones and side chain atoms. However, the function of Flvs with oligomeric protofibril is absolutely different compared to monomeric single chain Aβ, as shown in Fig. 4. Although Flvs tend to insert into the core of the protofibril, the secondary structure β-sheet content in oligomeric intermediate state remains almost intact (Figs. 3 and 4). It shows the break down of highly ordered and compact 3D structure of oligomeric U-shaped pre-nucleus pentameric β-strands by applying Flvs into irregular expanded intermediate states without dissociation. In order to calculate the interaction between A and B chains within U-shaped oligomeric peptides at fibril-like pentameric manner in the absence and presence of Flv compounds, we performed umbrella sampling simulations. Umbrella sampling is a robust method to obtain the ΔG_binding_ of a particular event along a reaction coordinate^29,30^. It can be seen in Fig. 5 that the chain A is entirely decomposed at 2.6 nm distance for the Flvs-free Aβ system, while the dissociated distance for the Flv-treated systems is greater than 3 nm. This indicates that the interaction of chain A with chain B in oligomeric protofibril structure is strengthened in the presence of Flvs, and as a result, the energy for separating the protofibril of chain A from B increases. The free energy of A:B binding is found to be − 38, − 40 and − 39 kcal/mol for Flv-free Aβ peptide and Aβ in complex with myricetin and morin Flvs, respectively. Therefore, Flvs are dislocated into the core of the protofibril as mainly interconnected chains through multi-functional groups that do not allow the adjacent peptide chains to be easily dissociated with respect to each other.

**Figure 6:**
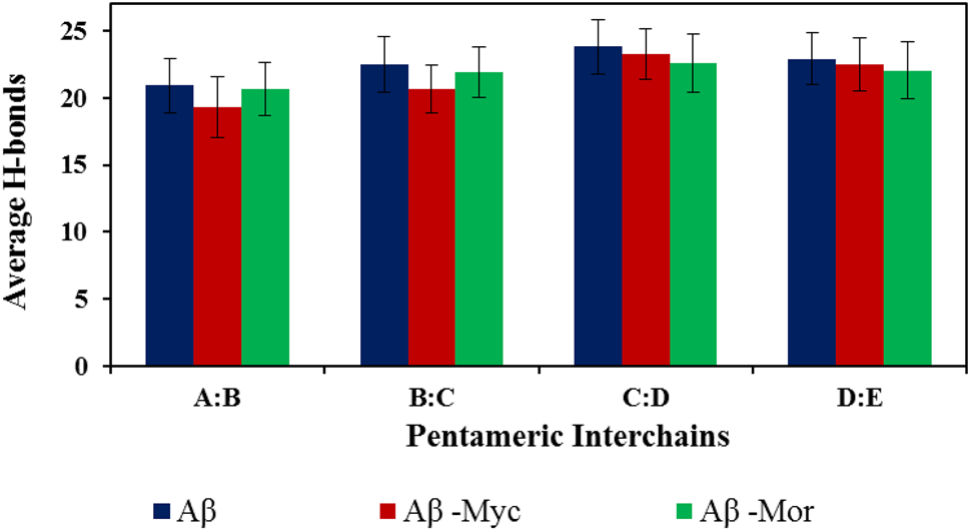
Average inter-chain backbone hydrogen bonds of pentameric protofibril with neighbor chains in the absence and presence of Flvs. The distance of 0.35 nm was assigned between H-bond donor and acceptor atoms to determine H-bond formation possibility^29^. All systems, including Flv-free Aβ, Aβ in complex with myricetin (Aβ-Myc) and morin (Aβ-Mor) Flvs are shown.

Moreover, analysis of inter-chain backbone hydrogen bonds of protofibril in the absence and presence of Flvs in Fig. 6 clearly denotes that inter-chain hydrogen bonds decrease by Flvs and can end in deformed oligomeric aggregates. The amyloid configuration and its stability depend on the number of hydrogen bonds involved between the backbone of the polypeptides inter-chains^36^. Due to possessing a number of OH groups, Flvs have the possibility of H-bond formation, both with the polypeptides’ backbone and side chain atoms of some residues. Therefore, the number of backbone hydrogen bonds between the chains decreased in complex with Flvs and these complexes progressively became loose and uncondensed aggregates, as shown in Fig. 4.

**Figure 7:**
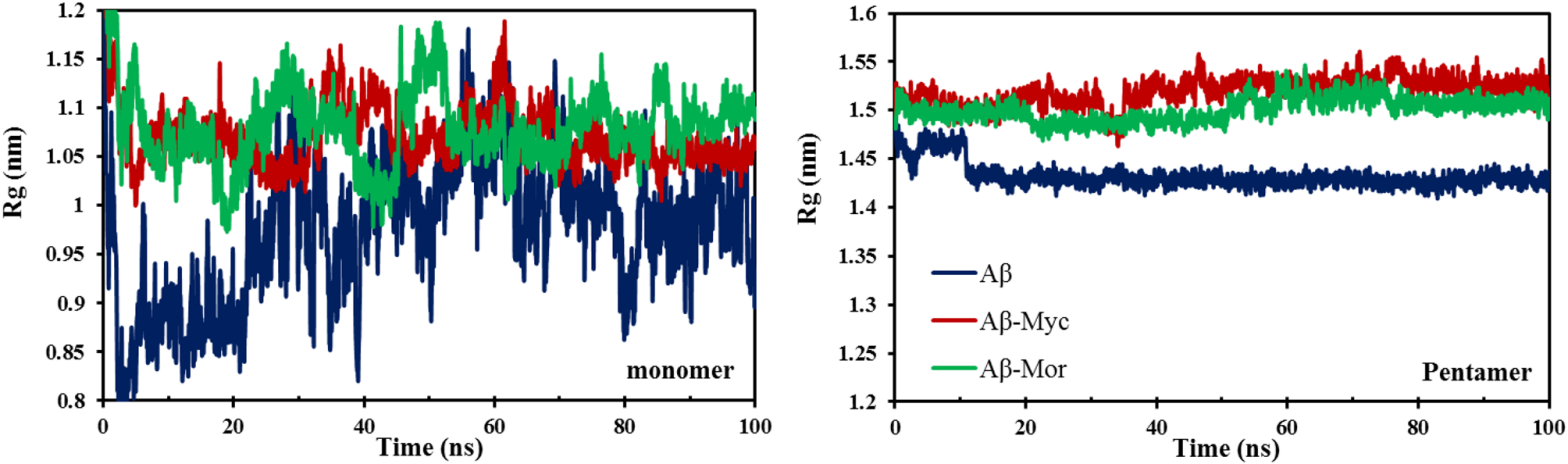
Radius gyration (Rg) of monomeric and pentameric protofibril of Aβ systems along 100 ns simulations. All systems, including Flv-free Aβ, Aβ in complex with myricetin (Aβ-Myc) and morin (Aβ-Mor) are shown.

On the other hand, analyses of radius of gyration (R_g_) as an indicator of compactness of protein structure (Fig. 7) clearly indicate the increasing R_g_ values for both Flv-Aβ complexes in monomeric and mainly pentameric systems. Highly increasing R_g_ values demonstrate an expanding conformation and so, it proves that the Aβ protofibril aggregates have been reoriented in the presence of Flvs. Therefore, since amyloid peptides in oligomeric intermediate aggregates are more compact and lead to Aβ fibrils by on-pathway mechanism, Flvs convert the condensed oligomeric aggregates to expand fragile nontoxic amorphous aggregates, which can prevent amyloid fibrils via off-pathway mechanism.

### Structural compactness of Aβ peptides based on mass density profile

**Figure 8:**
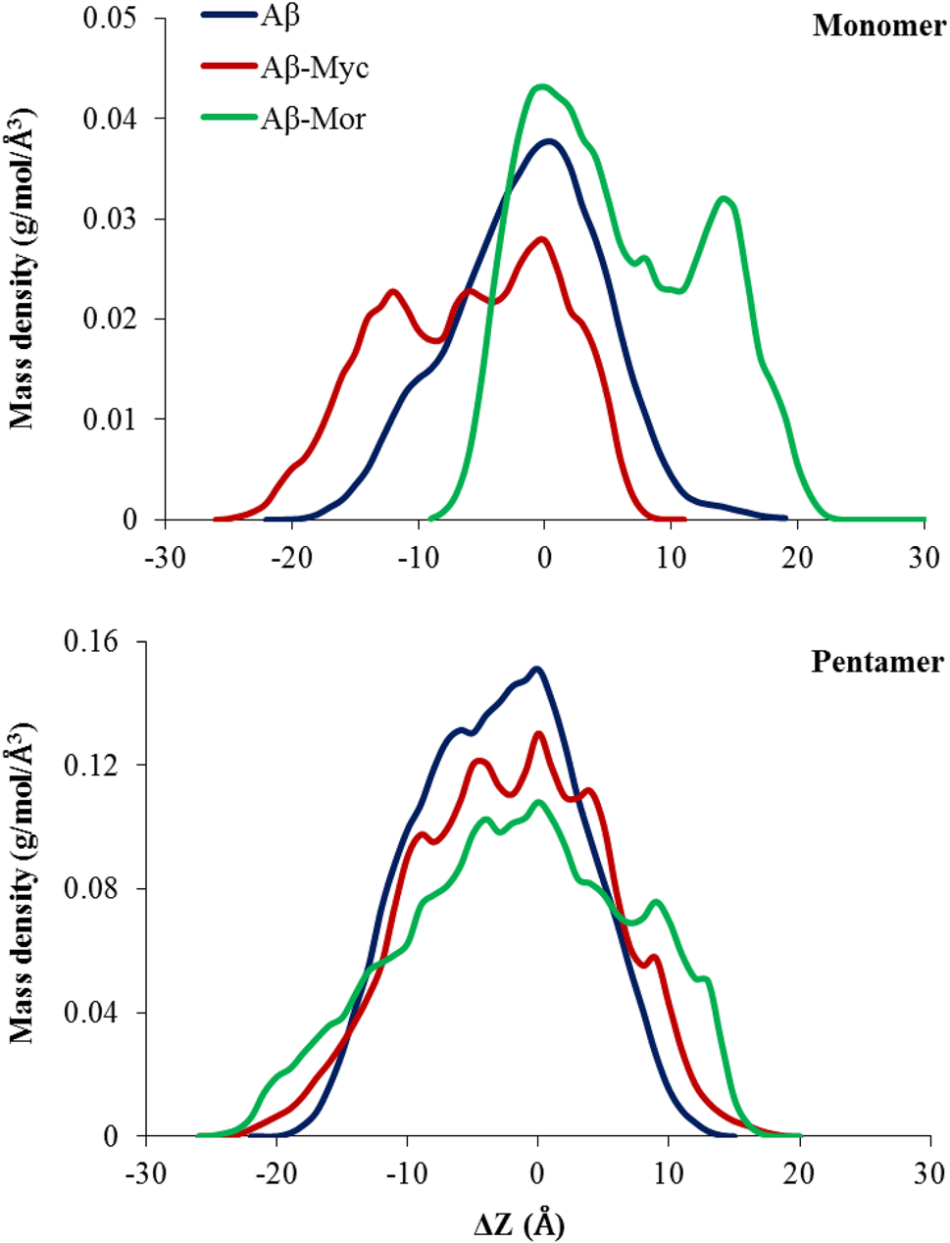
Mass density profiles for monomeric and pentameric protofibril of Aβ systems along the z-axis (resolution = 1 Å). The amount of |∆z| corresponds to the portions of thickness from dense core at ∆z ≈ 0. The distance from ∆z = 0 is a direct indicator of peptide width related to unfolded state. All systems, including Flv-free Aβ, Aβ in complex with myricetin (Aβ-Myc) and morin (Aβ-Mor) are shown.

To evaluate any possible change in structural density of the systems, 1-D projections of peptide mass density profile were analyzed and showed in Fig. 8. The Flv-free systems of monomeric and pentameric states display almost normal Gaussian distribution profile of mass density along the Z-axis. It means that Aβ structures in both monomeric and oligomeric states have similar structural density at the definite range of applied 1-D projections of Z-axis. The presence of Flvs resulted in both deviation of the Gaussian distribution profile and alteration of the mass density of peptides. Figure 8 clearly denotes that Flvs cause a decrease in mass density of peptides, expand the peptides thickness (increase in |∆z|) and emerge new peaks in both monomeric and pentameric systems. It means that the rigidity of Aβ peptides was obviously destroyed in complexed Flv-Aβ systems, especially in oligomeric state. Therefore, the previously discussed data are certainly confirmed by density profile assay, revealing that Flvs have dual functional capability against Aβ42 peptides.

### Resides inter-chain interaction analysis of oligomeric Aβ protofibril

**Figure 9:**
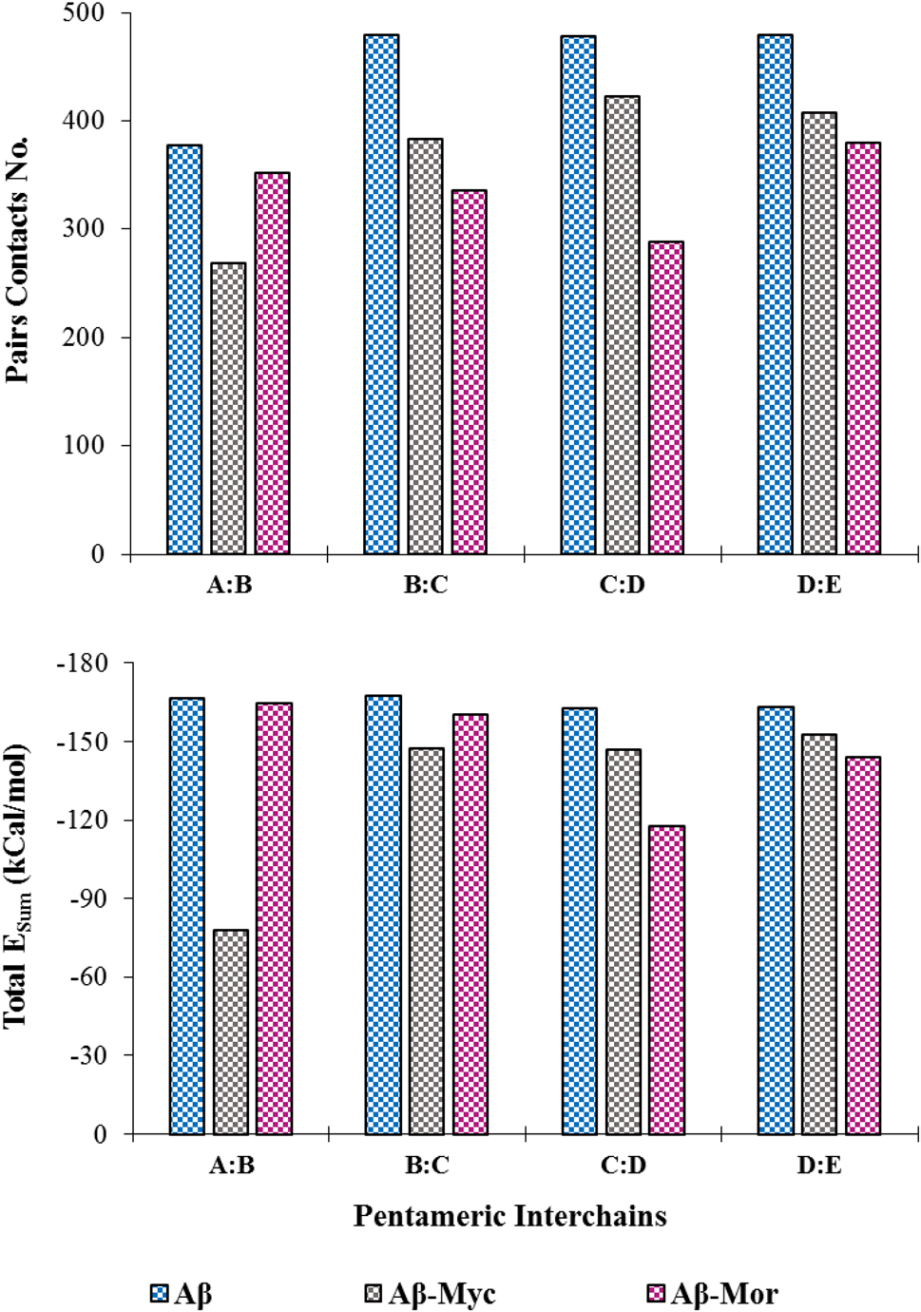
The number of total pairwise contacts (r < 5 Å) and the sum of total energies (E_sum_) of the related inter-chains of pentameric protofibril in the absence and presence of Flvs. 2500 MD simulation trajectories were used to extract the important residue interaction energy-based analysis. All systems, including Flv-free Aβ, Aβ in complex with myricetin (Aβ-Myc) and morin (Aβ-Mor) Flvs are shown.

**Figure 10:**
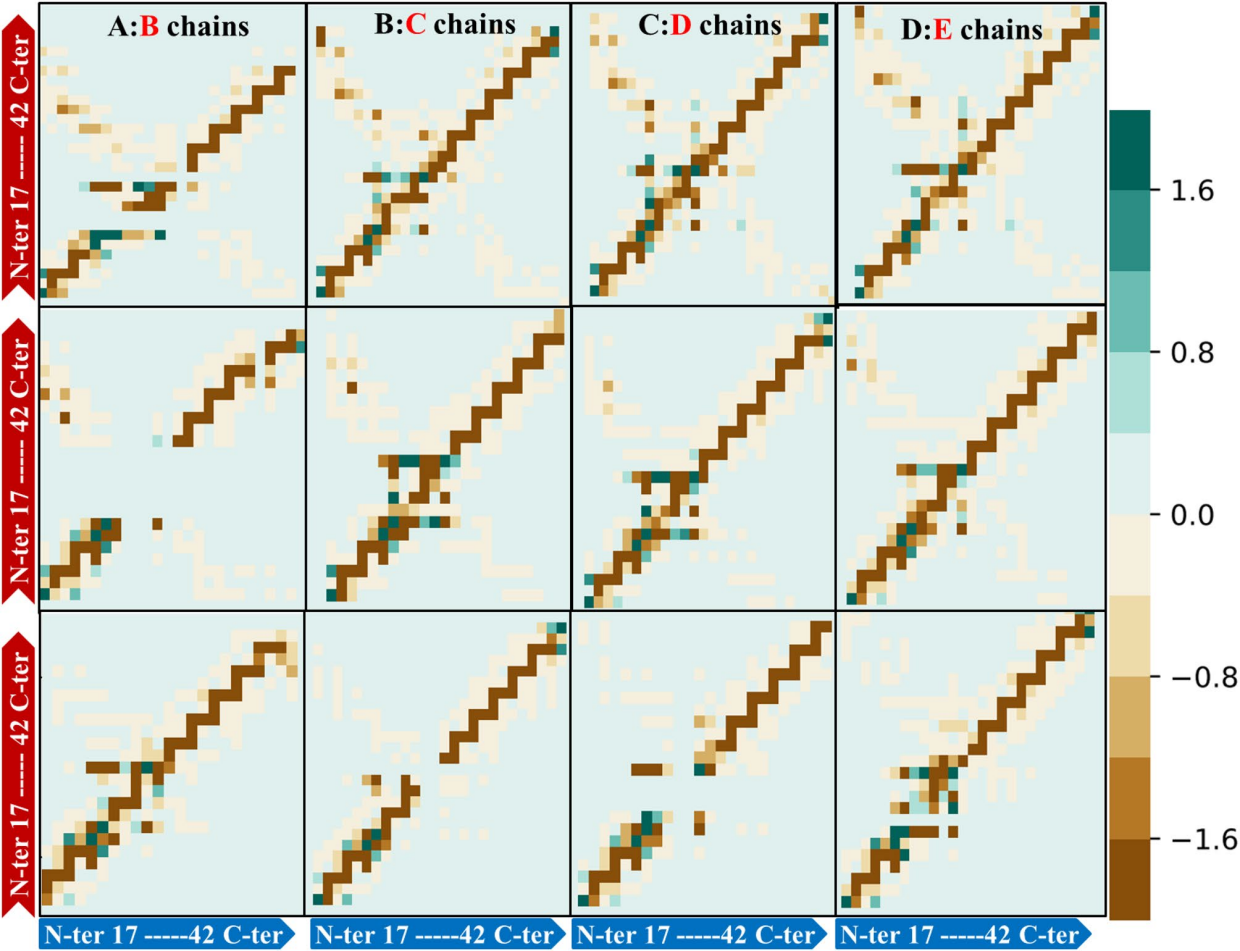
Interaction energy matrix (IEM) displays the energetic “hot-spot” contacts between residue pairs of adjacent chains in the pentameric protofibrils. The horizontal and vertical axes in each graph represent chains containing 17–42 residues, mentioned as A, B, C, D and E chains. Upper, middle and lower panels are related to Flv-free Aβ, Aβ in complex with myricetin and morin Flvs, respectively. The point-to-point higher favorite interactions between two neighbor chains are clearly shown as panels, in each the majority of plotted dots lie on the diagonal line, while the lateral interactions plot off the diagonal. Color range from negative to positive interaction energies are presented in kcal/mol.

We have developed gRINN tool to identify the crucial residue-residue interactions and to extract the energy network behavior from protofibril MD simulation trajectories^30^. Inter-chain amino acid non-bonded interaction energies for Aβ protofibril chains were computed and provided in Fig. 9 and Fig. S4. Data denoted the decrease in contact numbers with diminishing the pairwise inter-chain of residue-residue interaction energies in Flvs-treated systems, compared to the FLV-free one. The dynamical correlation of residue pairs interaction energies in the neighbor chains of pentameric Aβ protofibril was extracted as interaction energy matrix (IEM) in Fig. 10. The IEM shows that energetic “hot-spots” in the pentameric Aβ structure correspond to structure elements, which are not sequence neighbors but are in close interaction with each other in the 3D structure^31^. According to the color range and dot number, the strongest binding is shown on the panel diagonals, corresponding to homologous interactions between two neighbor chains with close contact, such as N- and C-terminal from one chain with similar part of the other neighbor chain. The dots plotted off the diagonal show the lateral interactions as non-homologous contacts related to N-terminal interactions of one chain with C- terminal of the other chain or vice versa. As shown in Fig. 10, the accumulation of hot-spots of interacted residues is higher in Flv-free system. The Flvs-treated systems demonstrate a decreased number of both homogenous and mainly non-homogenous hot-spot contacts between different chains of A, B, C, D and E in the pentameric protofibril intermediate. The strong total inter-chain interaction energies are related to electrostatic and van der Waals types, which result in compact oligomeric aggregates; however, both terms have been declined in Flvs-treated systems, as was already discussed. These findings are in good agreement with the previous results, where aggregated amyloid peptide is more compact in soluble state and lead to Aβ fibril by on-pathway mechanism^37^.

The important top 10 residue–residue interactions in each system with the lowest energy values are listed in Table 3. According to Table 3, some residue-residue interactions including (Asp23-Lys28, Phe20-Ala21, Phe20-Ala21, Phe19-Phe19, Ile31-Ile32, Val18-Phe19, and Asn27-Lys28) are maintained between each pair chain in all systems. Among these interactions, salt bridge interpeptide interaction between Asp23 with Lys28 is the most important one and plays crucial role in amyloid formation and protofibril stability^15^. Moreover, the salt bridge interaction in the flank sides of protofibril related to A:B and D:E was clearly attenuated by Flvs-treated systems. However, the other salt bridge in core region, including B:C, C;D remained almost intact despite the presence of Flvs. It means that Flvs are not able to dislocate the center core of U-shaped Aβ protofibril structure and to destroy the chains related to core region. They act efficiently on the surrounding flank chains of the salt bridge interactions by attenuating the pairwise salt bridge between A:B and D:E chains. Taken together, diminishing the total inter-chain interaction energies associated with electrostatic and van der Waals types attenuates the salt bridges in Flvs-treated systems, which end in fragile deformed oligomeric U-shaped protofilaments aggregates.

**Table 3:** Top 10 important inter-chain residue-residue interactions with the highest average total energies (E_total_) in pentameric form of Aβ peptide analyzed by gRINN tool.

Inter-chains	Aβ	E_total_	Aβ-Myc	E_total_	Aβ-Mor	E_total_
A:B	**ASP23-LYS28**	**− 60.3**	PHE20-PHE19	**− 4.99**	**ASP23-LYS28**	**− 40.86**
A:B	GLU22-LYS28	− 27.55	MET35-LEU34	− 4.7	**LYS28-ASN27**	− 8.83
A:B	LYS28-LYS28	− 8.94	GLY37-VAL36	− 4.59	VAL24-LYS28	− 7.63
A:B	VAL24-LYS28	− 8.23	**ILE31-ILE32**	− 4.56	SER26-ASN27	− 7.16
A:B	ASN27-SER26	− 6.36	**VAL18-PHE19**	− 4.48	SER26-LYS28	− 6.99
A:B	VAL18-PHE19	− 5.35	MET35-VAL36	− 4.44	PHE20-PHE19	− 5.39
A:B	**LYS28-ASN27**	− 5.3	GLY33-LEU34	− 4.4	VAL39-GLY38	− 5.35
A:B	**PHE20-ALA21**	− 5.09	**PHE20-ALA21**	− 4.38	ILE31-ILE32	− 4.8
A:B	ILE31-ILE32	− 4.88	PHE20-PHE20	− 4.28	MET35-LEU34	− 4.76
A:B	**PHE19-PHE19**	− 4.74	GLY33-ILE32	− 4.27	PHE20-PHE20	− 4.67
B:C	**ASP23-LYS28**	**− 69.81**	**ASP23-LYS28**	**− 71.54**	**ASP23-LYS28**	**− 69.32**
B:C	LEU17-ALA42	− 10.32	LYS28-ASP23	− 10.76	SER26-ASN27	− 5.92
B:C	LYS28-ASP23	− 7.01	VAL24-ASP23	− 9.81	ASN27-ASN27	− 5.65
B:C	**VAL18-PHE19**	− 5.15	**ASN27-LYS28**	− 9.58	PHE20-PHE19	− 5.26
B:C	**ASN27-LYS28**	− 5.09	ASN27-ASN27	− 7.09	SER26-LYS28	− 5.23
B:C	**ASN27-SER26**	− 4.8	**ILE31-ILE32**	− 4.78	MET35-LEU34	− 4.76
B:C	**PHE20-ALA21**	− 4.76	**VAL18-PHE19**	− 4.78	PHE20-PHE20	− 4.66
B:C	**PHE19-PHE19**	− 4.76	MET35-LEU34	− 4.75	ILE31-ALA30	− 4.65
B:C	MET35-LEU34	− 4.73	**PHE19-PHE19**	− 4.73	VAL24-GLY25	− 4.62
B:C	VAL24-ASP23	− 4.67	PHE20-PHE19	− 4.53	MET35-VAL36	− 4.26
C:D	**ASP23-LYS28**	**− 70.91**	**ASP23-LYS28**	**− 68.61**	**ASP23-LYS28**	**− 60.23**
C:D	LYS28-ASP23	− 7.62	LYS28-ASP23	− 9.86	VAL24-LYS28	− 10.98
C:D	ASN27-ASN27	− 5.98	VAL24-ASP23	− 7.48	PHE20-PHE19	− 5.34
C:D	MET35-LEU34	− 5.2	ASN27-ASN27	− 6.87	MET35-LEU34	− 4.76
C:D	**ASN27-LYS28**	− 5.09	**ASN27-LYS28**	− 6.55	PHE20-PHE20	− 4.7
C:D	**ILE31-ILE32**	− 4.81	**PHE19-PHE19**	− 4.79	**ILE31-ILE32**	− 4.55
C:D	**PHE19-PHE19**	− 4.74	PHE20-PHE19	− 4.69	ILE31-ALA30	− 4.46
C:D	**VAL18-PHE19**	− 4.67	SER26-ASN27	− 4.63	**PHE20-ALA21**	− 4.29
C:D	**PHE20-ALA21**	− 4.62	**VAL18-PHE19**	− 4.53	GLY33-ILE32	− 4.28
C:D	PHE20-PHE19	− 4.56	**ILE31-ILE32**	− 4.5	GLY33-LEU34	− 4.1
D:E	**ASP23-LYS28**	**− 64.08**	**ASP23-LYS28**	**− 45.74**	**ASP23-LYS28**	**− 40.7**
D:E	LEU17-ALA42	− 8.41	LYS28-ASP23	− 7.2	GLY25-ASP23	− 14.06
D:E	LYS28-ASP23	− 7.95	ASN27-ASN27	− 6.26	VAL24-ASP23	− 12.87
D:E	VAL24-LYS28	− 6.13	VAL24-ASP23	− 5.88	LYS28-ASP23	− 12.26
D:E	**ASN27-LYS28**	− 5.03	**ASN27-LYS28**	− 5.26	**ASN27-LYS28**	− 5.79
D:E	**PHE20-ALA21**	− 4.78	MET35-LEU34	− 4.87	PHE20-PHE19	− 5.17
D:E	**ILE31-ILE32**	− 4.73	**PHE20-ALA21**	− 4.62	MET35-LEU34	− 5.05
D:E	**PHE19-PHE19**	− 4.57	ILE31-ALA30	− 4.59	SER26-LYS28	− 4.91
D:E	PHE20-PHE19	− 4.54	SER26-ASN27	− 4.46	ILE31-ALA30	− 4.87
D:E	MET35-LEU34	− 4.44	PHE20-PHE19	− 4.46	PHE20-ALA21	− 4.49

2500 MD simulation trajectories were used to extract the average E_total_ for the Flvs-free Aβ, Aβ in complex with myricetin (Aβ-Myc) and morin (Aβ-Mor) Flvs. All energies are presented in kcal/mol.

### Inter-sheet structures of steric zipper analysis in oligomeric Aβ protofibril

Proteins and peptides known to assemble into amyloid fibrils have putative segments that form steric-zippers^38^. In amyloid fibrils, some segments are capable of forming a tight, dehydrated interface β-sheet structure. Steric zippers represent pairs of self-complementary β-sheets, which are formed by different and very short amyloid-forming segments^39^. The propensity of steric-zippers can be estimated from the self-complementary β-sheet sequences of each protein. The sequences of ^16^KLVFFA^21^ and ^37^GGVVIA^42^ segments have been identified as important self-complementary β-sheets for fibrillation in both Aβ40 and Aβ42^39,40^. In order to effectively investigate the disrupting propensities of Flvs on steric-zipper interactions, the van der Waals interaction energies between self-complementary residues of ^37^GGVVIA^42^ β-sheet segment were analyzed and shown in Table 4. The direct interaction energy evaluation of the self-complementary chain–chain residue pairs of steric zipper in oligomeric state of β-amyloid fibrils clearly indicates that the steric zipper contacts in Flvs-treated systems reduced during the simulation period. Since the intersheet steric zipper segments constitute the fibrils backbone and have an essential role in amyloid stability, disruption of the steric zipper contacts by Flvs could lead to structural destabilization of protofibrils. The steric zippers in the Aβ amyloid fibril provide key elements for the rational designation of inhibitors to prevent fibril formation. The aforementioned decrease in inter-chain interaction energies with attenuating H-bonds and salt bridges in Flvs-treated systems is concomitant with destabilization of steric zippers.

**Table 4:** The steric zippers, pairs of self-complementary β-sheets, related to the key amyloid-forming segments with sequences of 37–42 (^37^GGVVIA^42^) were analyzed for possible van der Waals interaction energies in different systems.

Inter-chains	E_Gly37-Gly37_	E_Gly38-Gly38_	E_Val39-Val39_	E_Val40-Val40_	E_Ile41-Ile41_	E_Ala42-Ala42_
A:B Aβ_free_	− 0.778	− 0.731	− 1.26	− 1.14	Null	Null
A:B Aβ-Myc	− 0.63	Null	− 0.148	Null	Null	Null
A:B Aβ-Mor	− 0.544	− 0.668	− 0.427	Null	Null	Null
C:D Aβ_free_	− 0.753	− 0.745	− 1.28	− 1.2	− 1.17	− 0.199
C:D Aβ-Myc	− 0.743	− 0.75	− 1.23	− 1.12	− 0.835	− 0.29
C:D Aβ-Mor	− 0.694	− 0.615	− 1.29	− 1.2	Null	Null
B:C Aβ_free_	− 0.753	− 0.762	− 1.28	− 1.31	− 1.5	− 0.823
B:C Aβ-Myc	− 0.74	− 0.557	− 0.94	Null	Null	Null
B:C Aβ-Mor	− 0.677	− 0.707	− 1.22	− 1.16	− 0.86	− 0.305
D:E Aβ-_free_	− 0.597	− 0.647	− 1.18	− 1.3	− 1.35	− 0.665
D:E Aβ-Myc	− 0.779	− 0.676	− 1.2	− 1.2	− 1.41	Null
D:E Aβ-Mor	− 0.719	− 0.669	− 1.27	Null	Null	Null

Flvs effectively disrupt the steric-zipper interactions related to contact segment of ^37^GGVVIA^42^ by decreasing or losing the self-complementary Waals interaction energies in the associated pair of inter-chains β-sheets. The systems of Aβ_free_, Aβ-Myc and Aβ-Mor are denoted for pentemeric Aβ peptides in Flvs-free state, treated with myricetin and morin Flvs, respectively. All energies are presented in kcal/mol.

### Mechanism of Flvs dual function against Aβ42 aggregation and fibrillogenesis

**Figure 11:**
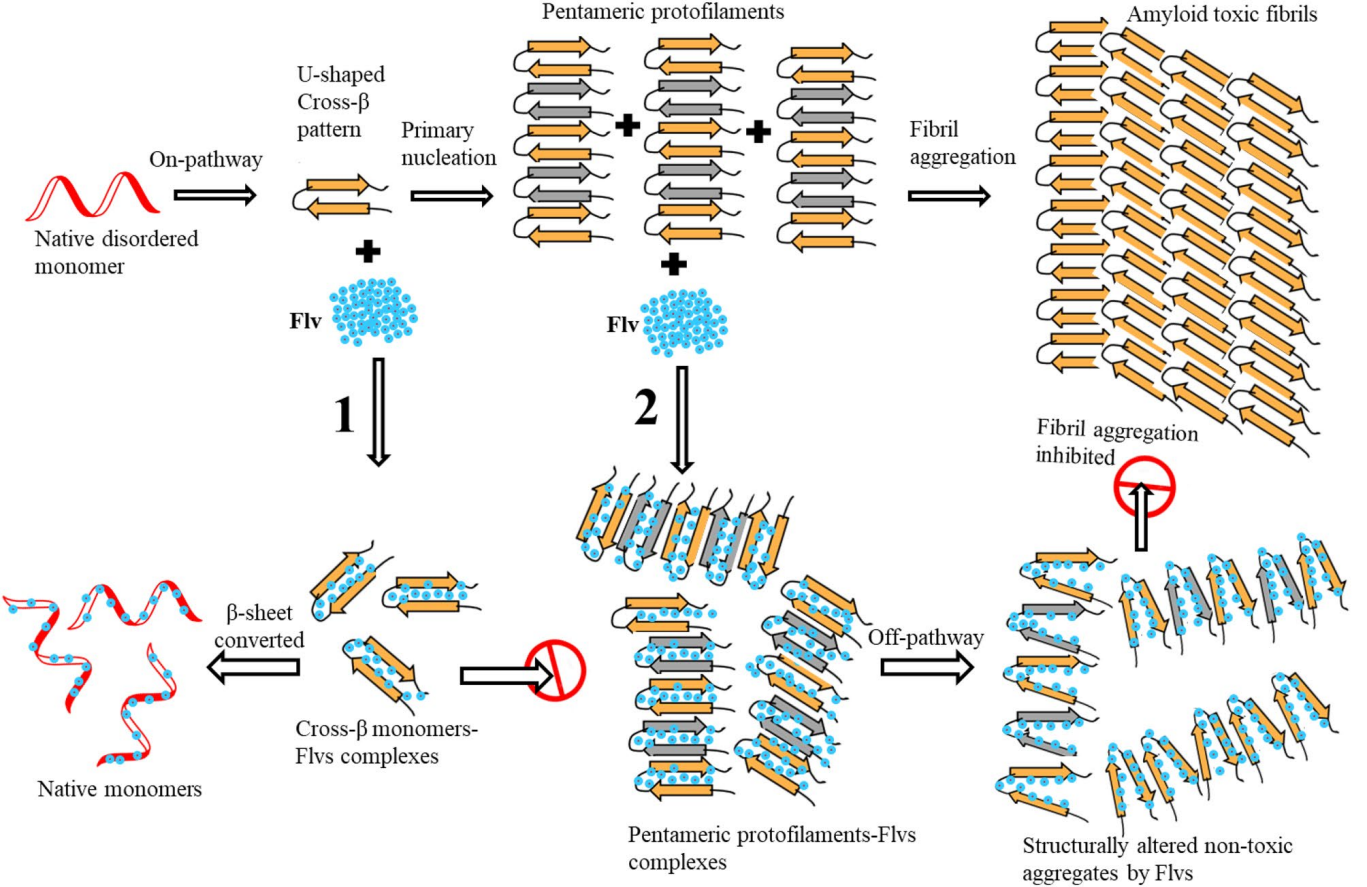
Schematic illustration for the action mechanism of polyphenolic flavonoids (Flv) on the inhibition of Aβ fibrillogenesis and modulation of Aβ aggregation by off-pathway mechanism. The favorable dual role of Flvs is related to fibrillogenesis suppression (pathway **1**) and modulation of Aβ aggregation by off-pathway mechanism (pathway **2**) with the formation of non-toxic aggregates. Protein misfolding undergoes conformational change into U-shaped β-strands, followed by primary nucleation to form pentameric protofilaments that end in mature amyloid toxic fibrils via on-pathway mechanism. On-pathway mechanism breaks down by Flv in two intermediate states as U-shaped β-strands and pentameric protofilaments; the misfolded monomers form irregular β-hairpin monomers and result in aggregated oligomers, which do not end in fibrils. Flvs interact with U-shaped β-strand monomers in pre-nucleation and pentameric protofilament states that result in fibrillogenesis suppression (pathway **1**) and modulation of Aβ aggregation by off-pathway mechanism (pathway **2**), respectively.

The presence of secondary structure β-sheet content in amyloidogenic intermediate state is a crucial step in progressing Aβ aggregates. We found that the Flv compounds act as inhibitors for fibrillogenesis through completely converting monomeric APS β-sheet structures into coil/helix. However, the compounds only deform pre-nucleus pentameric U-shaped aggregates as protofilament intermediates without interfering with their β-sheet contents. Oligomeric intermediate aggregates could end in amyloid fibrils via on-pathway mechanism or nontoxic amorphous aggregates by off-pathway mechanism; both pathways are associated with 3D aggregated conformers. Based on the discussed data, the action mechanism of Flvs was clearly depicted in Fig. 11 with schematic representation. The utmost important process related to the conversion of secondary β-sheet structures into coil or helical features clearly occurs as an essential early step at aggregation-prone states during prevention of amyloid fibrillation by Flv compounds (Pathway 1). The coil elements of Aβ42-Flv prevent both the primary nucleation and the fibril aggregation. In addition, it was found and clearly represented in Fig. 11 that Flvs deform the highly ordered β-sheet structure of oligomeric Aβ42 pentamer as an essential pre-nucleus intermediate step in Aβ amyloidogenesis through on-pathway mechanism, resulting in the redirect off-pathway aggregation process (Pathway 2). Note that the main common part in both processes is the nucleation, since the nucleus aggregates are present in both on and off-pathway aggregation processes. However, on-pathway oligomers end in toxic fibril components of amyloid plaques through highly ordered nuclei with secondary β-sheet structures. Off-pathway oligomers lack regular structures of each monomer in the related nuclei and end in irregular non-toxic β-aggregates (do not end in fibrils). Fortunately, both the non-specific pathway and off-pathway process end in fibrillogenesis inhibition. Therefore, dual functions of Flvs, including suppression of fibrillogenesis and deformation of oligomeric protofilament intermediate of fibrils were clearly achieved by applying two monomeric and pentameric systems.

## Conclusion

The role of natural Flv molecules in destabilization of amyloid fibril aggregates has been identified as a promising approach for treating AD. However, the mechanism underlying the inhibition of Aβ fibril formation by polyphenol is least elucidated. Our data demonstrated that Flvs directly bind to Aβ structure in both monomeric and pre-nucleus aggregates with independent functions of: a) transition from β-strand to random coil structure in monomeric APS of Aβ through a non-specific pathway (the disadvantageous contents of secondary β-sheet conformations in monomeric Aβ have been completely disappeared in myricetin and morin Flvs-treated systems), and b) remodeling the oligomeric pre-nucleus Aβ aggregation pathway towards the formation of off-pathway unstructured fragile aggregates through disrupting the steric zipper motif of fibrils related to the pairs of self-complementary β-sheets in pre-nucleus aggregates. Therefore, we can conclude that Alzheimer's risk may reduce by applying Flv-rich food in very early stage of amyloid-beta formation at the aggregation-prone state, in order to convert any possible β-sheet peptides without fibril formation.

## Supplementary information

Supplementary Information.
