# Supplementary material for: Simulations on the dual effects of flavonoids as suppressors of Aβ42 fibrillogenesis and destabilizers of mature fibrils
Source: Sci Rep. 2020 Oct 6;10:16636. doi: 10.1038/s41598-020-72734-9 (PMC7538952; doi:10.1038/s41598-020-72734-9)
Supplement: Supplementary file 1 — Supplementary Information. [file 41598_2020_72734_MOESM1_ESM.docx]

**Simulations on the dual effects of flavonoids as suppressors of Aβ42 fibrillogenesis and destabilizers of mature fibrils**

Sahar Andarzi Gargari*^1^, Abolfazl Barzegar**^1,2^

*^1^Research Institute of Bioscience and Biotechnology, University of Tabriz, Tabriz, Iran*

*^2^Department of Medical Biotechnology, Faculty of Advanced Medical Sciences, Tabriz University of Medical Sciences, Tabriz, Iran*

**Corresponding author: [barzegar@tabrizu.ac.ir](mailto:barzegar@tabrizu.ac.ir)

*Co-corresponding author: [s](mailto:tarinejad@azaruniv.edu)ahar_andarzi@yahoo.com

Tel: +98-41133393926

Fax: +98-4113294113

**Fig. S1.** Presenting the primary sequence and tertiary U-shape rod-like structure of Aβ peptide pentamer. Core region of peptide consist of N-terminal β-strand (termed β1, residues 18-26), C-terminal β-strand (β2, residues 31-42), with a turn region connecting the two strands.


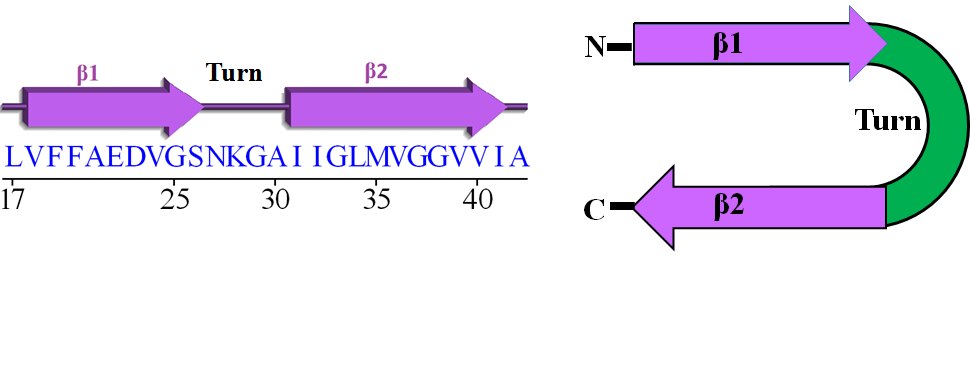

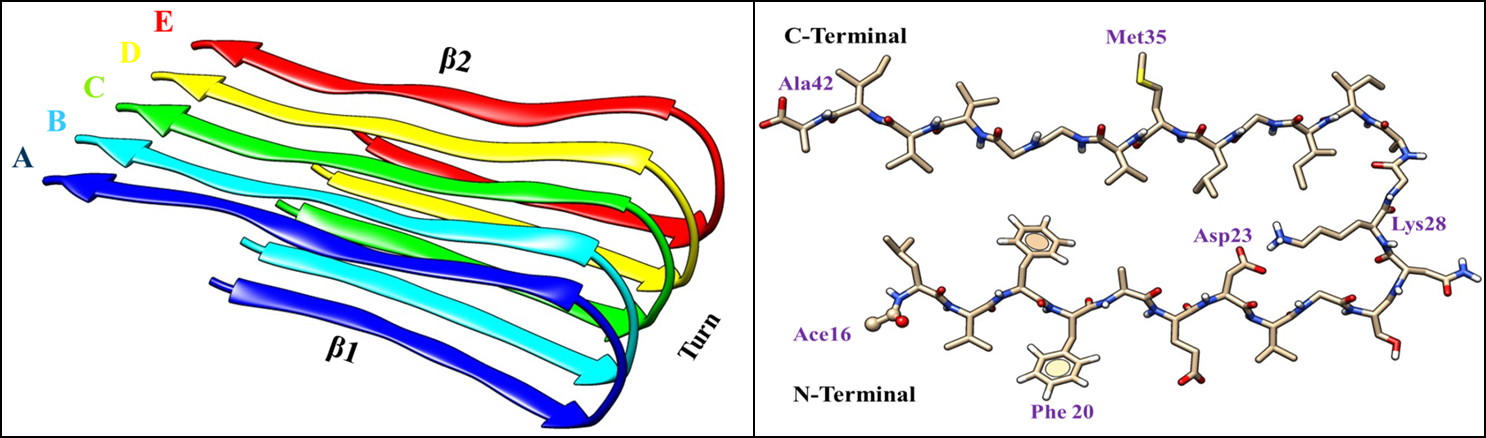


**Fig. S2.** DFT optimized and two-dimensional structures of Myricetin and Morin flavonoids. Myricetin with 6 functional OH groups and Morin with 5 functional OH groups can make hydrogen bonds with Aβ peptide.

**
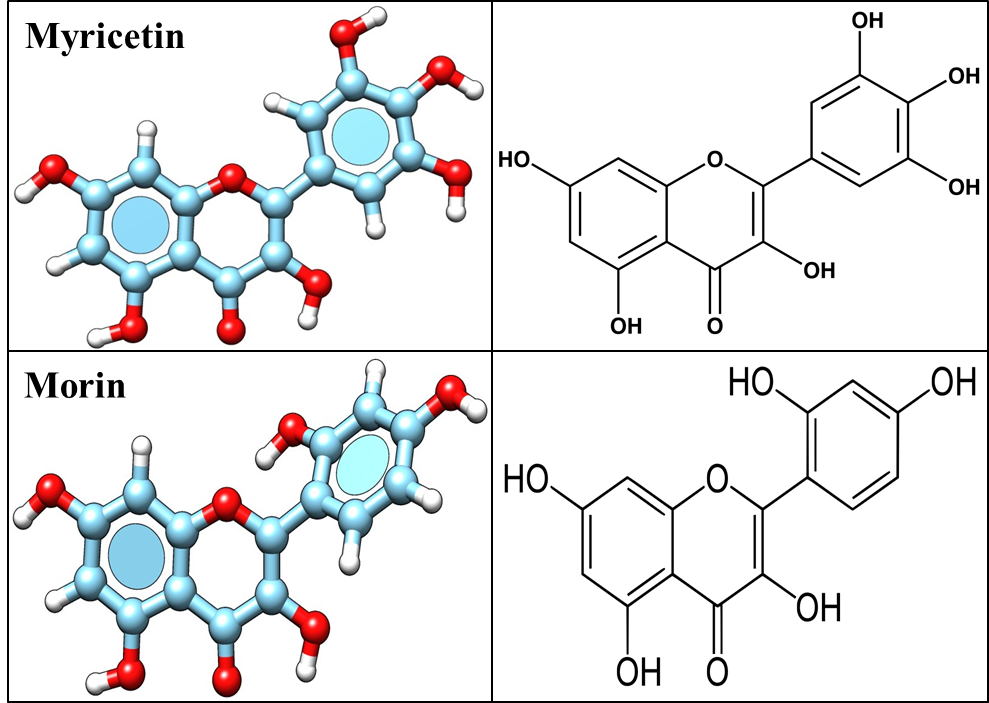
**

**Fig. S3.** The percentage of β-sheet conformations as a function of the Aβ42 monomer residues.


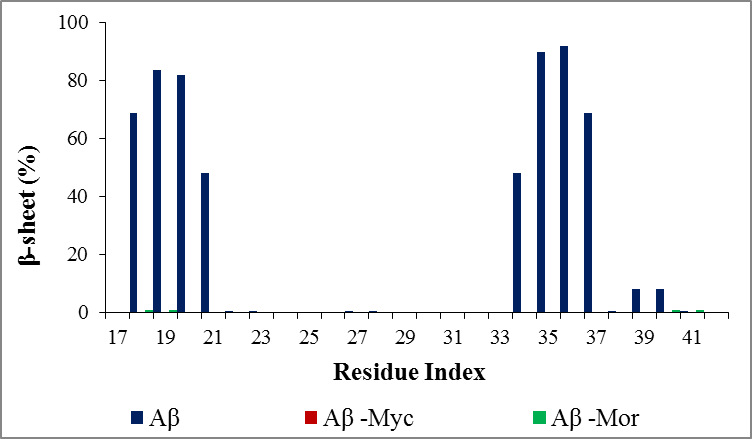


**Fig. S4.** Sum of interaction energies in terms of electrostatic and van der Waals types of free Aβ protofibril (green), Aβ in complex with myricetin (red), Aβ in complex with morin (black).

**
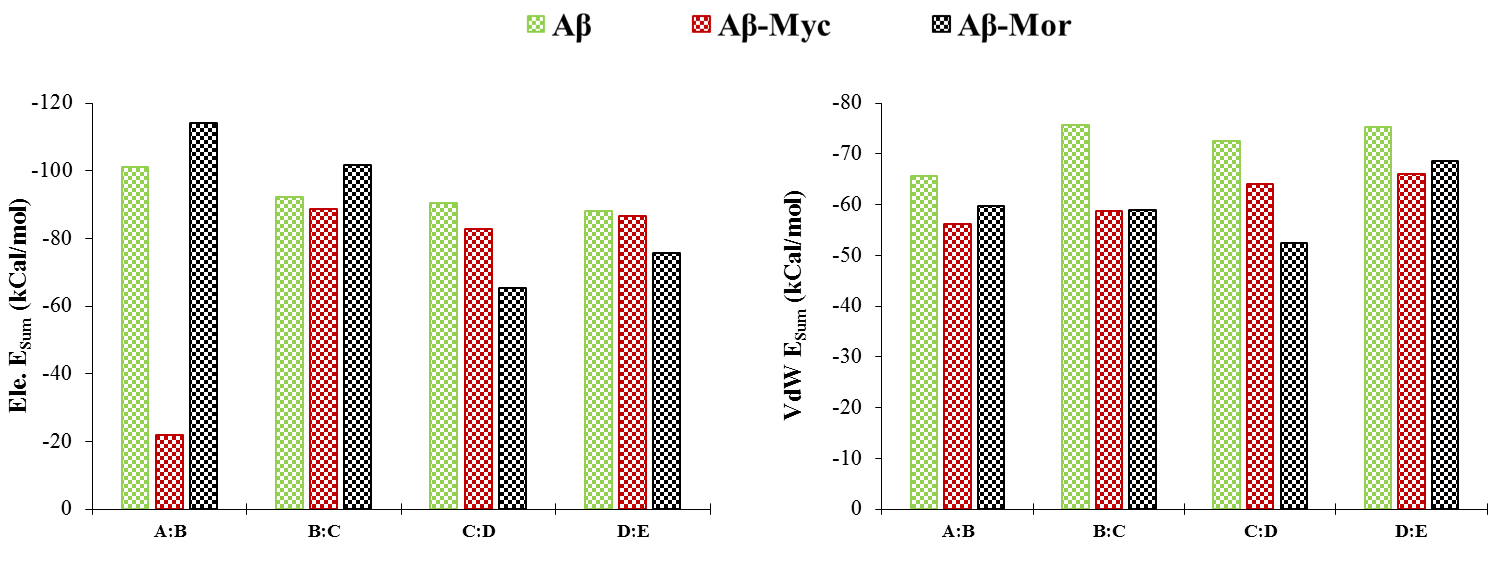
**
